# Bronchial Atresia of the Right Ninth Bronchus with Segmental Hyperinflation in an Asymptomatic Adult—A Case

**DOI:** 10.3390/diagnostics16131966

**Published:** 2026-06-24

**Authors:** Wolfgang Jungraithmayr, Birte Ohm, Jakob Neubauer

**Affiliations:** 1 Division of Thoracic Surgery, Rostock University Medical Center, Schillingallee 35, 18057 Rostock, Germany; 2 Department of Thoracic Surgery, Medical Faculty, RWTH Aachen University, 52074 Aachen, Germany; 3 Department of Diagnostic and Interventional Radiology, Medical Center—University of Freiburg, Faculty of Medicine, University of Freiburg, 79106 Freiburg, Germany

**Keywords:** bronchial, atresia, hyperinflation, emphysema

## Abstract

Bronchial atresia (BA) is a rare congenital anomaly that develops as a consequence of an intrauterine bronchial artery insult. Distal to the atresia, a mucocele can form with consecutive hyperinflation of the peripheral lung parenchyma. We describe an asymptomatic patient with a well-demarcated segmental emphysematous area within the right lower lobe revealed by computed tomography (CT). Here, the right lateral basal segmental bronchus (B9) is proximally interrupted while the distal, mucus-filled bronchus forms a bronchocele. The down-stream segmental parenchyma is hyperinflated. 3D reconstruction of the tracheobronchial tree reveals a normal architecture of the tracheobronchial tree except for the characteristic discontinuation of the right ninth bronchus. Asymptomatic patients with BA do not require treatment, however, follow-up CT is recommended to assess stability of the segmental hyperinflation.

**Figure 1:**
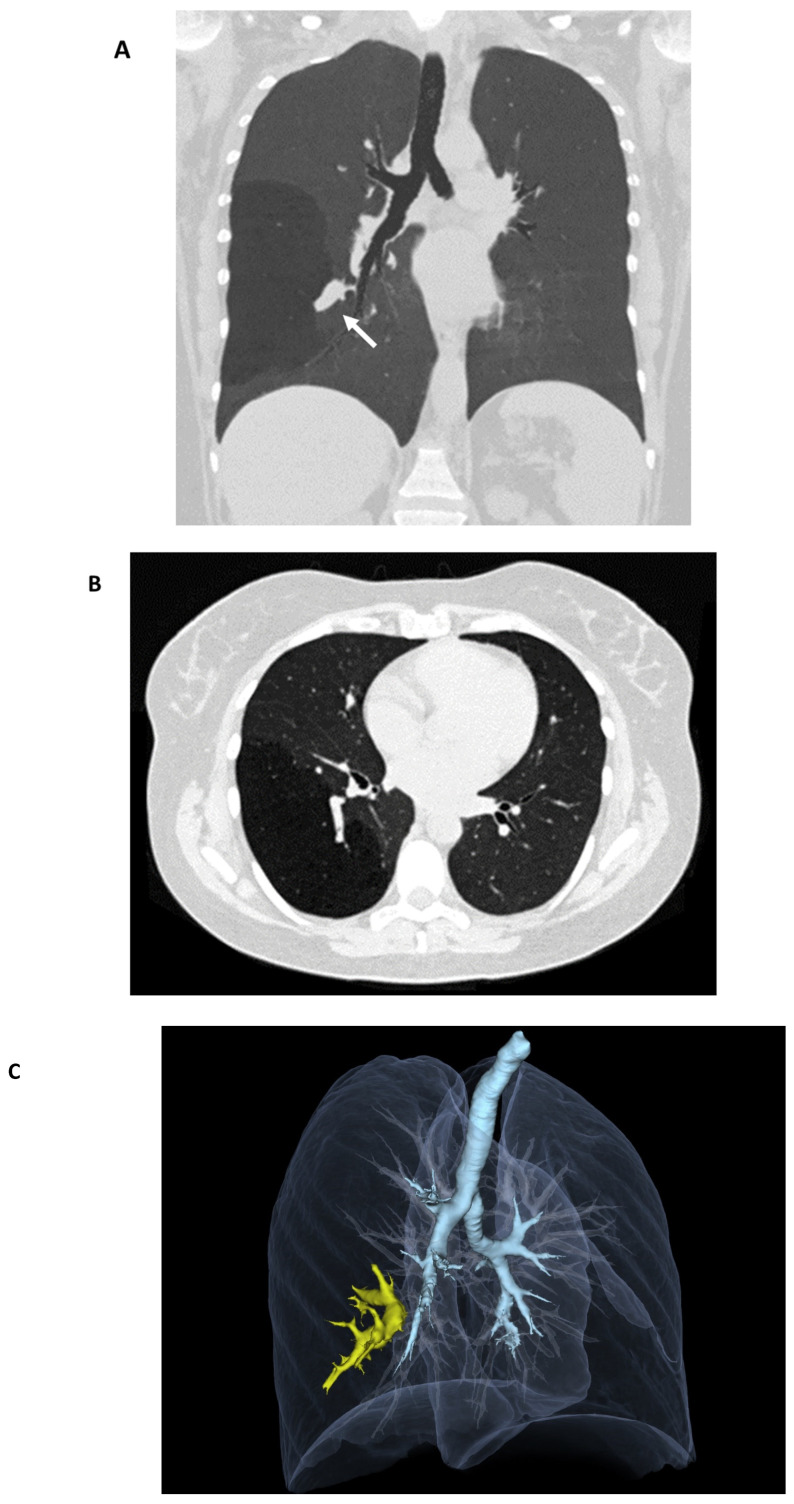
Bronchial atresia (BA) is a rare congenital disorder and usually evolves as a consequence of a bronchial artery occlusion or an intrauterine infection during the prenatal period [1]. Typically, segmental bronchi are affected. Distal to the atresia, a mucocele can develop. As a consequence, the lung parenchyma distal to the affected bronchus is hyperinflated. This is caused by collateral ventilation through intra-alveolar pores of Kohn. BA is typically found in the upper lobes [2,3]. (**A**,**B**) The asymptomatic patient presented here had a computed tomography (CT) which revealed a well-circumscribed emphysema of the right lower lobe as an incidental finding. (**A**,**B**) shows a complete obstruction of the right lateral-basal segmental bronchus (B9). The proximal part of the segmental bronchus is obliterated while the distal part is dilated and filled with fluid consistent with a bronchocele ((**A**), white arrow). (**C**) Three-dimensional volume-rendered CT reconstruction of the tracheobronchial tree and lungs depicts the patent central and segmental bronchi in light blue, while the isolated mucus-filled distal B9 bronchus, the bronchocele, is highlighted in yellow. The semi-transparent lung overlay provides anatomic orientation. At the expected origin of the right lower-lobe B9 segmental bronchus, the continuity with the proximal bronchial tree is missing, demonstrating the bronchial interruption which is characteristic for bronchial atresia. Surgical treatment of BA is only required if the patient becomes symptomatic. However, a follow-up CT scan is recommended to assess for progression of the hyperinflation.

## Data Availability

The original contributions presented in this study are included in the article. Further inquiries can be directed to the corresponding author.
